# Addressing elder abuse through integrating law into health: What do allied health professionals at a Community Health Service in Melbourne, Australia, think?

**DOI:** 10.1111/ajag.12720

**Published:** 2019-09-17

**Authors:** Virginia J. Lewis, Vanessa White, Faith Hawthorne, Jess Eastwood, Robyn Mullins

**Affiliations:** ^1^ Australian Institute of Primary Care and Ageing La Trobe University Bundoora Victoria Australia; ^2^ Justice Connect Melbourne Victoria Australia; ^3^ Cohealth Melbourne Victoria Australia

**Keywords:** Allied health personnel, elder abuse, elder neglect, health care

## Abstract

This research looked at the attitudes of Community Health Service (CHS) staff regarding the integration of a lawyer into their CHS both before and after the integration occurred. It assessed their confidence in identifying and addressing elder abuse at each point. A written survey was distributed to staff before the lawyer commenced (n = 126), and approximately 12 months afterwards (n = 54). The preliminary survey demonstrated widespread agreement that legal issues can affect older people and supported having a lawyer in a CHS. Respondents were not confident about their capacity to identify abuse and provide referrals to a lawyer, but this improved in the follow‐up survey. These CHS staff were aware of the potential impacts of elder abuse and supported embedding a lawyer in the health service. Information and training as part of this service model should focus on the skills needed for CHS staff to play their role in such a partnership.

**Policy Impact**

Health Justice Partnerships are increasingly popular as a model for improving access to legal services. Allied health professionals working with older people experiencing elder abuse or at risk or abuse would welcome the support of a lawyer. Health services should consider the inclusion of a lawyer when establishing integrated health services.

**Practice Impact**

Lawyers would be a welcome addition in health services that are dealing with older people suffering from abuse or at risk of abuse. Health professionals do not need convincing of the potential benefits of working with a lawyer, but do need training, information and/or support to have the skills and confidence to recognise and act on suspected elder abuse.

## 1 INTRODUCTION

Elder abuse has been defined by the World Health Organization as ‘a single or repeated act, or lack of appropriate action, occurring within any relationship where there is an expectation of trust, which causes harm or distress to an older person. It can be of various forms: physical, psychological/ emotional, sexual, financial or simply reflect intentional or unintentional neglect’ (pg3).^1^ The impacts of elder abuse can include depression, anxiety, post‐traumatic stress disorder (PTSD), poor self‐reported health,^2^ involuntary relocation to an aged care facility and even increased mortality.^3^

It is difficult to determine the extent of the prevalence of elder abuse, as there is no agreed definition of what constitutes abuse, what types of abuse should be measured and at what age someone should be considered to be an ‘elder’. Data collection is also difficult when members of the affected group may be unwilling or unable to speak out.^4^ Some Australian reports cite an elder abuse prevalence of 2.7%‐4.6%.^5^ However, other studies have estimated higher rates: a recent US review, based on three population studies, estimated that approximately 10% of those over 60 living in the community are abused to some degree.^6^ An international review estimated an average incidence of 14.3%.^7^ One study estimated that there were 24 unknown cases of elder abuse for every one that was known.^8^

As with estimates of the extent of elder abuse, it is difficult to estimate what types of abuse are the most common. In Victoria, an analysis of calls over a two‐year period to a helpline run by Seniors Rights found that 60% of callers were experiencing issues classified as ‘elder abuse’. Financial abuse and psychological/emotional abuse were the most common forms of abuse, with women the most likely victims and adult children the most common perpetrators.^9^ Although these people may not be typical, in that they were seeking help, information or advice about elder abuse, the data do give some insight into the types of difficulties experienced by older people.

Interventions to prevent or stop elder abuse vary and may focus on assisting the abused person or helping the perpetrator change their behaviour, but few high‐quality studies have assessed these interventions’ effectiveness.^10^ This is particularly the case for adults who live in the community, as opposed to those in institutions, where efforts to change the behaviour of paid carers are often the aim of the intervention.^11^

Older people who are being abused may be reluctant to seek help for many reasons, including not recognising their situation as abuse and/or being dependent on the perpetrator. However, they are very likely to attend or receive services for health care; approximately 95% of people over 65 see a general practitioner at least once a year and many also see allied health professionals in the same period.^12^ This contact means primary health care workers are ideally placed to identify people experiencing elder abuse. However, they may not necessarily have the skills or means to recognise elder abuse or to act on any concerns. Many frontline health and community workers who identify abuse do not have organisational policies or strategies on how to address it,^13^ which can leave them feeling stressed and unsupported.^14^

Collaboration between health and legal professionals is one way to ensure legal issues are addressed for older people who would not necessarily actively seek out legal solutions to their difficulties. Well‐timed interventions can also help prevent relatively minor issues from escalating to the point of abuse. One solution is for health professionals to refer consumers/clients to legal services, including Community Legal (CL) services.

Victoria has a large, well‐established community health (CH) sector, which provides universal access to health services, as well as targeted services for vulnerable population groups. Services offered are wide‐ranging and may include the following: medical and nursing care; dental health; and physiotherapy, podiatry, occupational therapy, alcohol and drug programs and disability services. There has been a strong positive relationship between the CH and CL sectors in Victoria for many years, given they service similar priority populations, comprising disadvantaged people with limited resources.

Justice Connect, a not‐for‐profit CL service, has a Health Justice Partnership (HJP) with cohealth—a multisite community health service (CHS). The HJP aims to offer an integrated service that will extend legal support to older consumers of the CHS who may be experiencing, or are at risk of, elder abuse.^15^ The two organisations had a collaborative approach to determining the aims and objectives of the HJP, with senior managers from each organisation meeting regularly with the HJP lawyer to ensure consistency in operational management. Governance arrangements were formalised, and an Advisory Group (with representatives from government, experts in family violence responses of health services, pro bono lawyers, seniors’ rights advocates and a philanthropic representative) provided strategic oversight and support. Developing policies for dealing with issues around client information and confidentiality was particularly important. More details about the development of the HJP are available online.^16^

The lawyer was on‐site initially at one location for four days per week, later spreading their time over three locations over a three‐year period. This was a deliberate strategy, as both formal (team meetings, professional development, newsletters) and informal (coffee catch‐ups, corridor conversations) engagement was crucial to the success of the HJP.

The lawyer provided single‐session information and training to staff working in areas of the health service that the CHS advised were most likely to have contact with older people, including intake, the Aged Residential and Outreach Team, homelessness, community health, and the Hospital Admission Risk Program. Sessions provided information about the issues that might affect older people that could have legal solutions, and how to identify them. Staff were told that they could approach the lawyer with general or specific questions, seek information on behalf of a client or ask the client if they would like to meet the lawyer. Staff could contact the lawyer directly or seek an appointment for a client by phone, by email or in person.

As a new model of service delivery, an integrated legal service must be accepted by health professionals for it to be successful. Although staff at CHSs are accustomed to working as part of a multidisciplinary care team, it was not known how they feel about working with lawyers, particularly on issues around elder abuse.

This paper reports on a survey of the attitudes health professionals held about integrating a lawyer into a health service before the project commenced and approximately one year after it had been in place. It also reports on their confidence in identifying and addressing elder abuse. The survey was part of an overall evaluation of the HJP—details of service uptake will be reported elsewhere.

## 2 METHODS

The surveys were designed by the authors with additional input from the Project Advisory Group and senior health service staff. The survey was not piloted beyond the group involved in its development; however, it was reviewed by members of the health service's internal ethics advisory group, which includes managers, staff and consumers. The surveys were designed to be completed before the lawyer commenced taking referrals at a specific site and approximately one year after the lawyer began at the specific site. Surveys were distributed to staff by email (by a manager) and in person (by the lawyer) at baseline, and then again in the same way one year after the lawyer commenced taking referrals at that site; thus, data collection was spread over time. Consent was implied by completion of the survey, and no incentives or reminders were used. Questions addressed the following: respondents’ general feeling about law and health professionals working together; more specific questions about the law, health and older people; and finally, how comfortable and confident staff felt about working with older people who possibly needed legal help. The health service elected not to identify the respondents; therefore, rather than being treated as repeated measures for individual health professionals, the data at each time point are an indication of the support of the staff at the level of the health service teams.

### 2.1 Ethics

The study had ethics approval from cohealth and as a project with negligible risk from the La Trobe University College of Science, Health and Engineering Human Research Ethics Committee (S16‐200).

## 3 RESULTS

Participants comprised 127 people who responded to the survey before the lawyer was embedded in the service site and 54 who responded at follow‐up. The data were not matched, but at follow‐up, 20 respondents indicated they had completed the baseline survey, 26 had not and 8 could not recall.

Questions were divided into attitudes and beliefs about the links between law and health (Table 1) and personal confidence in identifying and dealing with abuse. Staff were asked the extent to which they agreed with a series of statements relating to law and health and responded on a 6‐point scale from *Disagree strongly* to *Agree strongly, or Don't know*. Given the high number of empty cells and the number of cells with expected counts of less than 5, non‐parametric Mann‐Whitney U tests were conducted to compare the responses at the two time points. All missing and *Don't know* responses were excluded from this analysis.

**Table 1:** Attitudes to integrating law and health (%)

		Agree Strongly	Agree Moderately	Agree Slightly	Disagree Strongly	Disagree Moderately	Disagree Strongly	Mann‐Whitney *U*
It is a good idea to have a lawyer as part of a community health service	Pre	70.9	16.2	9.4	1.7	0.9	0.9	*P*<.01
	Post	90.2	7.8	2.0	0.0	0.0	0.0	
The health of older people can be negatively affected by legal problems and issues	Pre	77.9	14.8	5.7	0.0	0.8	0.8	ns
	Post	80.0	16.0	0.0	4.0	0.0	0.0	
Receiving help with legal problems and issues can improve the health of older people	Pre	67.8	24.0	6.6	0.8	0.0	0.8	ns
	Post	81.1	17.0	1.9	0.0	0.0	0.0	
Older people experience issues and problems (financial and emotional) that could be addressed with legal solutions	Pre	50.0	34.4	12.3	2.5	0.0	0.8	ns
	Post	53.8	38.5	5.8	1.9	0.0	0.0	
Health professionals have a role to play in addressing elder abuse^a^	Pre	74.1	18.1	6.0	0.9	0.0	0.9	ns
	Post	82.7	15.4	1.9	0.0	0.0	0.0	
Legal and health professionals should work together in addressing elder abuse^a^	Pre	71.6	21.6	6.0	0.0	0.0	1.0	ns
	Post	82.7	11.5	5.8	0.0	0.0	0.0	

a Participants were instructed to use the ‘don't know’ category if they did not know what ‘elder abuse’ was, so are excluded from the analyses.

As the data in Table 1 show, even at baseline there was near‐universal agreement that there were legal solutions to some of the problems older people face; and that health professionals alone, and health professionals and lawyers working together, should address elder abuse. The only item to which responses changed significantly between pre‐ and post‐test was the belief it was a good idea to have a lawyer in a community health service, where agreement became even stronger.

As the data in Table 2 indicate, prior to having any information about or experience of the HJP, respondents lacked confidence about their ability to identify abuse and then potentially refer clients to a lawyer, including some who indicated they were not sure what ‘elder abuse’ referred to. After the service had been in operation for 12 months, those responding felt more confident in all aspects of their ability to identify abuse.

**Table 2:** Personal confidence in identifying and addressing abuse (%)

		Agree Strongly	Agree Moderately	Agree Slightly	Disagree Strongly	Disagree Moderately	Disagree Strongly	Mann‐Whitney *U*
I am confident I can identify problems that older people experience that could be addressed by consulting a lawyer	Pre	13.4	26.9	31.1	15.1	10.9	2.5	*P*<.001
	Post	23.1	51.9	17.3	3.8	3.8	0.0	
I feel comfortable asking older people questions that would reveal if they are being abused emotionally or are being neglected	Pre	20.8	35.8	22.5	9.2	7.5	4.2	*P*<.001
	Post	28.6	57.1	12.2	2.0	0.0	0.0	
I am confident I have the skills and knowledge to refer clients to a lawyer	Pre	14.4	23.7	26.3	13.6	11.9	10.2	*P*<.001
	Post	34.6	46.2	9.6	3.8	5.8	0.0	
I feel comfortable asking older people questions that would reveal whether they are experiencing financial abuse	Pre	17.6	28.0	28.8	12.0	8.0	5.6	*P*<.001
	Post	41.5	37.7	11.3	3.8	5.7	0.0	
I am confident I can identify whether an older person is experiencing elder abuse^a^	Pre	67.8	24.0	6.6	0.8	0.0	0.8	*P*<.01.
	Post	81.1	17.0	1.9	0.0	0.0	0.0	

a Participants were instructed to use the ‘don't know’ category if they did not know what ‘elder abuse’ was, so are excluded from the analyses.

Of the 54 people who responded to the follow‐up survey, 39 had attended an information session with the lawyer. People who had attended a session were more likely to have had a secondary consultation with the lawyer (23 attendees, three non‐attendees, *χ* ^2^ = 5.81, *P* = .02). Attendees were also more likely to have referred someone to the lawyer than non‐attendees (14 cf 1, *χ* ^2^ = 4.19, *P* = .04).

## 4 DISCUSSION

Health Justice Partnerships (or Medical‐Legal Partnerships in the United States) have been adopted widely in efforts to address social disparities in health. Interest in developing such partnerships has been demonstrated at an organisational level in services for homeless people.^17^ However, little is known of the views of the health‐care professionals themselves. Nor are older people a common focus of such partnerships, although the levels of disadvantage and legal issues they face suggest they should be.^18^ Our study sought to determine health‐care professionals’ views on integrating law and health services, with a focus on older people and elder abuse, both before and after a lawyer began working at their centre.

Support for the general idea of having a lawyer as part of a community health service increased significantly after the HJP model had been in place for 12 months. This is an encouraging finding, as staff are unlikely to change their behaviour and utilise the new service if they do not perceive that a lawyer could help their clients.

Respondents were also very positive about the intersection of law and health, specifically as it related to older people or elder abuse; and although there were no significant changes after experiencing the HJP model, responses shifted consistently towards more positive attitudes, comprising mainly an increase in the strength of agreement from ‘slight or moderate agreement’ to ‘strong agreement’. The only point staff members were reserved about was the potential benefits of legal solutions related to ‘financial and emotional’ problems. It is not clear why support was lower for this than for other seemingly similar items, but it was the only item where ‘emotional’ problems were mentioned as an issue. Possibly, CHS staff are less convinced that ‘emotional’ issues could have legal solutions than the other issues mentioned in the survey.

Prior to the HJP being implemented, many respondents doubted their capacity to identify elder abuse and deal with it. On all the measures of personal confidence about identifying abuse and addressing it, confidence increased, probably as a result of receiving training/information, the experience of being around the lawyer or seeing the impact of the lawyer's involvement with clients.

Respondents who had attended a session with the lawyer were more likely to have sought out a secondary consultation or to have referred a client to the lawyer. This points to the importance of offering information sessions, in addition to providing informal ways for the lawyer and health‐care staff to interact.

The research was limited by the smaller sample for the follow‐up survey, and it is possible that the people who chose to respond were staff who were the most enthusiastic about the HJP. It is also possible that some staff chose to participate because the HJP was most relevant to their clients. The numbers of staff in each team who were invited to complete a survey were not documented, so it was not possible to calculate response rates. These limitations do not negate the findings from the preliminary survey, which indicated that while staff held positive attitudes towards having a lawyer in the CHS, belief in their own skills was more limited. This work was carried out in Victoria, where there is a history of community health and legal services cooperating in various ways to address social inequity. In addition, an outreach clinic staffed by pro bono lawyers had previously operated at one site of the health service, albeit without achieving the intended reach.^19^ These experiences may have predisposed staff to be positive about the role of the law in health.

Findings strongly suggest that staff in this community health service were already on board with the general idea of integrating a lawyer into the service and saw the benefits of having a lawyer available for older consumers. This may not be the case in other services, where staff are not as accustomed to working with professionals from other disciplines.

## 5 CONCLUSION

A baseline survey such as the one conducted here is a useful place to start, to determine the attitudes of the staff. In this instance, information sessions, training and any other efforts to encourage staff to work with a lawyer did not need to focus on the benefits, but on upskilling staff so they were confident about identifying abuse and had the practical skills to know how and when to involve the lawyer.

## CONFLICT OF INTEREST

The authors declare no conflict of interest.

## References

[1] World Health Organization . The Toronto Declaration on the Global Prevention of Elder Abuse. 2002. https://www.who.int/ageing/projects/elder_abuse/alc_toronto_declaration_en.pdf Accessed August 15, 2019.

[2] Acierno R , Hernandez‐Tejada MA , Anetzberger GJ , Loew D , Muzzy W . The National Elder Mistreatment Study: An 8‐year longitudinal study of outcomes. J Elder Abuse Negl. 2017; 29(4): 254‐269. 28837418 10.1080/08946566.2017.1365031

[3] Dong XQ , Simon MA , Beck TT , et al. Elder abuse and mortality: The role of psychological and social wellbeing. Gerontology. 2011; 57(6): 549‐558. 21124009 10.1159/000321881 PMC5546614

[4] Moir E , Blundell B , Clare J , Clare M . Best practice for estimating elder abuse prevalence in Australia: moving towards the dynamic concept of adults at risk and away from arbitrary age cut‐offs. Current Issues Crim Just. 2017; 29(2): 181‐190.

[5] Kurrle S , Naughtin G . An overview of elder abuse and neglect in Australia. J Elder Abuse Negl. 2008; 20(2): 108‐125. 18928212 10.1080/08946560801974521

[6] Lachs M , Pillemer K . Elder abuse. N Engl J Med. 2015; 373(20): 1947‐1956. 26559573 10.1056/NEJMra1404688

[7] Pillemer K , Burnes D , Riffin C , Lachs MS . Elder abuse: global situation, risk factors, and prevention strategies. Gerontologist. 2016;56(Suppl_2): S194‐S205. 26994260 10.1093/geront/gnw004 PMC5291158

[8] Lifespan of Greater Rochester, Inc., Weill Cornell Medical Center of Cornell University New York City Department for the Aging . Under the Radar: New York State Elder Abuse Prevalence Study. https://ocfs.ny.gov/main/reports/Under%20the%20Radar%2005%2012%2011%20final%20report.pdf. Accessed April 19, 2019.

[9] Joosten M , Dow B , Blakey J . Profile of elder abuse in Victoria: analysis of data about people seeking help from Seniors Rights Victoria. National Ageing Research Institute in partnership with Seniors Rights Victoria;2015.

[10] Fearing G , Sheppard CL , McDonald L , Beaulieu M , Hitzig SL . A systematic review on community‐based interventions for elder abuse and neglect. J Elder Abuse Neglect. 2017; 29(2–3): 102‐133. 10.1080/08946566.2017.1308286 28339321

[11] Ayalon L , Lev S , Green O , Nevo U . A systematic review and meta‐analysis of interventions designed to prevent or stop elder maltreatment. Age Ageing. 2016; 45(2): 216‐227. 26744361 10.1093/ageing/afv193

[12] Australian Bureau of Statistics 2017 . Health Service Usage and Health Related Actions, Australia, 2014‐15 4364.0.55.002 *.* http://www.abs.gov.au/AUSSTATS/abs@.nsf/allprimarymainfeatures/CD8DB1EFA25F665DCA257B39000F371A?opendocument Accessed April 18, 2019.

[13] Ahmed A , Choo W‐Y , Othman S , et al. Understanding of elder abuse and neglect among health care professionals in Malaysia: an exploratory survey. J Elder Abuse Negl. 2016; 28(3): 163‐177. 27149412 10.1080/08946566.2016.1185985

[14] Cairns J , Vreugdenhil A . Working at the frontline in cases of elder abuse: 'it keeps me awake at night'. Australas J Ageing. 2014; 33(1): 59. 24521488 10.1111/ajag.12017

[15] Hawthorne F , Eastwood J . Working together: a health justice partnership to address elder abuse. Elder Law Rev. 2016;10: 1‐3.

[16] Justice Connect . Health Justice Partnerships. https://justiceconnect.org.au/our-services/seniors-law/about-hjps/. Accessed May 22, 2019.

[17] Tsai J , Jenkins D , Lawton E . Civil legal services and medical‐legal partnerships needed by the homeless population: a national survey. Am J Public Health. 2017; 107(3): 398‐401. 28103065 10.2105/AJPH.2016.303596 PMC5296685

[18] Tyler Tobin L . Socially vulnerable older adults and medical‐legal partnership. National Center for Medical Legal Partnership, 2019 https://medical-legalpartnership.org/wp-content/uploads/2019/03/Socially-Vulnerable-Older-Adults-and-MLP.pdf Accessed 30 May, 2019.

[19] Lewis V , Adamson L , Hawthorne F . Health justice partnerships: a promising model for increasing access to justice in health services. Aust Health Rev. 2018; 42(5): 1A‐1C. 30196803 10.1071/AH18101

